# ID 268 - Fluxograma de Seleção de Estudos Automatizada pelo Direct_Flow: estudo descritivo

**DOI:** 10.5327/2237-9622.2025.v34s1.268

**Published:** 2025-11-25

**Authors:** Isabela Porto de Toledo, Rafael Leite Pacheco, Ana Luiza Cabrera Martimbianco, Carolina de Oliveira Cruz Latorraca, Veronica Colpani, Isabela Porto de Toledo, Roberta Borges Silva, Camila Cruz Monteiro, Cecília Menezes Farinasso, Patrícia do Carmo Silva Parreira, Rachel Riera

**Keywords:** síntese de evidências, fluxograma do processo de seleção dos estudos, Stata

## Abstract

**Introdução:**

Uma etapa fundamental de toda síntese de evidências é a descrição do processo de seleção dos estudos. Esta descrição é idealmente realizada por meio de fluxogramas, que devem conter os resultados das buscas e o número de estudos eliminados na primeira fase de seleção, de estudos excluídos na segunda fase de seleção e de estudos incluídos.

**Método:**

Estudo descritivo da elaboração do programa direct_flow desenvolvido na linguagem de programação ado do software Stata. Esse programa foi desenvolvido no contexto de dois projetos conduzidos pelo Núcleo de Avaliação de Tecnologias em Saúde/Núcleo de Evidências do Hospital Sírio-Libanês (Nats/NEv-HSL) em colaboração com o Ministério da Saúde, a Agência Nacional de Saúde Suplementar e o Conselho de Nacional de Justiça, por meio do Programa de Apoio ao Desenvolvimento Institucional do Sistema Único de Saúde (Proadi-SUS). Ambos os projetos envolvem a elaboração de sínteses de evidências, e o desenvolvimento do direct_flow visa à elaboração de fluxogramas automatizados para aumentar a eficiência e a consistência entre as sínteses.

**Resultados:**

O programa direct_flow funciona por meio de pré-programados, que podem ser escolhidos pelo pesquisador. Foram elaborados 11, dos quais 7 são específicos para revisões sistemáticas e 4 para de revisões sistemáticas. De modo geral, o pesquisador precisa escolher o e indicar, em comandos padronizados, o número de referências em cada fase do processo de seleção. As caixas são preenchidas automaticamente e figuras são geradas em alta resolução. Todos os possuem versões em português e inglês. O programa é de download gratuito via Stata e mais detalhes, além de todos os , podem ser encontrados em https://rlpacheco.github.io/direct_flow/.

**Conclusão:**

A elaboração de sínteses de evidências pode ser complexa e consumir bastante tempo. A automatização na geração de figuras e outros passos pode reduzir a carga de trabalho e garantir qualidade e consistência entre diferentes sínteses. Programas automatizados nos mais diversos softwares podem facilitar etapas em todas as revisões sistemáticas. O direct_flow foi uma ferramenta criada no âmbito de dois projetos financiados pelo Proadi-SUS, mas que poderá ser utilizado de maneira aberta por todos os Nats da Rebrats.

